# A Telephone-Adapted Mindfulness-Based Stress Reduction Program: Preliminary Effects among Healthcare Employees

**DOI:** 10.3390/bs11100139

**Published:** 2021-10-18

**Authors:** Lauren A. Zimmaro, Aleeze Moss, Diane K. Reibel, Elizabeth A. Handorf, Jennifer B. Reese, Carolyn Y. Fang

**Affiliations:** 1 Cancer Prevention and Control Program, Fox Chase Cancer Center, Philadelphia, PA 19111, USA; lauren.zimmaro@fccc.edu (L.A.Z.); elizabeth.handorf@fccc.edu (E.A.H.); jennifer.reese@fccc.edu (J.B.R.); 2 Myrna Brind Center for Mindfulness, Marcus Institute of Integrative Health—Jefferson Health, Department of Integrative Medicine and Nutritional Sciences, Sidney Kimmel Medical College, Thomas Jefferson University, Philadelphia, PA 19107, USA; aleeze.moss@jefferson.edu (A.M.); diane.reibel@jefferson.edu (D.K.R.)

**Keywords:** mindfulness, mindfulness-based stress reduction, telephone adaptation, telehealth, healthcare employees, distress, depression, anxiety, self-compassion

## Abstract

Healthcare employees often experience high stress and may benefit from accessible psychosocial interventions. In this pilot study, we explored preliminary feasibility, acceptability, and psychological effects of a telephone-based adaption of mindfulness-based stress reduction (MBSR) for healthcare employees. Eleven participants (M age = 49.9; 27.3% ethnic/racial minority) were enrolled in an eight-session group-based MBSR program adapted for telephone delivery. Feasibility was assessed using rates of program attrition and session completion; acceptability was explored qualitatively via participants’ responses to an open-ended item about their program experience. Participants also completed pre-and post-program assessments on psychosocial outcomes (distress (overall distress, depression, anxiety, somatization), mindfulness, and self-compassion). We characterized mean change scores, 95% confidence intervals, and effect sizes to explore preliminary program effects. With regard to preliminary feasibility, one participant dropped out prior to the intervention; of the remaining 10 participants, 90% completed at least half (≥4) of the sessions; 70% completed at least three-quarters (≥6 sessions). Feedback reflected positive experiences and included suggestions for program delivery. Participants reported reductions in distress post-program (M difference range = −5.0 to −9.4), showing medium to large effect sizes (*d* range = 0.68 to 1.11). Mindfulness scores increased from pre- to post-intervention (M difference range = 1.0 to 10.4), with small-to-medium effects (*d* range = 0.18 to 0.55). Almost all aspects of self-compassion remained stable over time, with the exception of common humanity, which increased post-program (M difference = 2.9, CI 95% 0.5 to 5.4, *d* = 0.91). Preliminary findings from our small pilot trial suggest that telephone-based adaptations of MBSR may be a useful mode of delivery for healthcare employees; however, larger studies are needed to provide further evidence of feasibility, acceptability, and program effects.

## 1. Introduction

Stress is common among healthcare employees [1,2,3], with rates of emotional distress and burnout increasing in recent years [4]. Given the substantial physical and emotional demands involved with providing and supporting high-quality patient care, it is unsurprising that healthcare employees can experience significant levels of anxiety, depression, and compassion fatigue [1,2,3,4,5,6,7,8]. High distress among healthcare employees is not only linked to poorer individual health outcomes [8], but also to poorer work performance and patient care and outcomes as well [8,9,10,11]. Thus, addressing healthcare employee stress and psychological well-being is a critical issue that has garnered increasing attention [5,6,12]. 

To address stress and psychological well-being among healthcare employees, a number of workplace-based interventions have been developed and evaluated. One popular program is mindfulness-based stress reduction (MBSR). Mindfulness is defined as paying attention to the present moment purposefully and non-judgmentally [13,14,15]. The MBSR program as originally designed consisted of eight instructor-led weekly sessions held in a small-group format in-person, with each session lasting approximately 2.5 h [13,14,15]. The program was designed to teach participants how to improve their ability to cope with the stresses of daily life through practicing mindfulness. In line with theoretical models of mindfulness for coping with distress (including feelings of stress, anxiety, and depression) [16], mindfulness is thought to promote positive psychological and behavioral change by supporting a non-judgmental awareness of one’s experiences. As such, one closely related aspect of mindfulness is self-compassion, which is the ability to bring awareness, warmth, and understanding to oneself and perceived inadequacies. [17]

Mindfulness has been shown to be an effective approach for decreasing distress and improving well-being across a wide variety of clinical and non-clinical populations [18,19,20]. Within healthcare settings in particular, MBSR programs can be applied to teach healthcare employees how to better manage chronic occupational stress in order to avoid or reverse symptoms of professional burnout [21,22]. Indeed, such programs have been shown to be beneficial for healthcare employees’ symptoms of stress [21,22,23,24] as well as for quality of patient care [25].

Despite evidence for the benefits of MBSR programs among healthcare employees, many of these programs report high attrition and drop-out rates [21,23]. For example, one study cited a 44% intervention drop-out rate as compared to wait-list controls [26]. This may be due in part to barriers and other issues associated with the in-person format of MBSR. Many healthcare employees work long hours or have rotating shifts, which constrains one’s ability to attend regular in-person programs [5]. In addition, healthcare employees may feel sensitive about the lack of anonymity when engaging in a personal growth or therapy program among work colleagues [5]. However, few studies have examined the utility of an adapted MBSR program that could address these challenges in a healthcare setting, where privacy and convenience might be heavily valued. For example, adapting the MBSR program to a telephone-based format, rather than an in-person format, might offer significant benefits, including (1) enhancing ease of participating from one’s home, instead of within the work setting, (2) increasing the flexibility of scheduling and attending sessions (without the added stress of travel, etc.), and (3) providing a more anonymous format, which may facilitate participants’ engagement with less concern for confidentiality. 

Therefore, the aims of the current study were to explore the preliminary feasibility, acceptability, and psychological effects of an adapted eight-week telephone-based MBSR program among employees in a healthcare setting. We hypothesized that the program would be feasible and acceptable, as explored through session completion rates and open-ended written participant feedback, respectively. Although our outcomes were exploratory, we hypothesized that there would be preliminary evidence of improved psychological outcomes (decreased distress, increased mindfulness and self-compassion) as described through pre- to post-program change on these measures and corresponding effect sizes. If successful, our study could demonstrate the potential benefits of offering an MBSR-based intervention via telephone in populations where this modality may be more appropriate than in-person sessions, such as among healthcare employees. 

## 2. Materials and Methods

### 2.1. Participants and Procedures

Participants were recruited from a comprehensive cancer center in an urban mid-Atlantic city using flyers posted in public areas. Information about the program was also distributed via an electronic news bulletin that was sent by email to employees. Inclusion criteria included: (1) age 18 or older and (2) able to communicate in English. Exclusion criteria included: (1) Self-reported diagnosis of a psychotic disorder or major dissociative disorder and (2) presence of a cognitive deficit that would make it difficult to participate in an eight-week telephone program or complete the brief study measures. Individuals who were interested in participating contacted study staff for more information. After ascertaining eligibility, written informed consent was obtained by study staff. Enrolled participants then completed a baseline assessment (see Section 2.3 Measures), were provided with program materials, and participated in the eight-week program (see Section 2.2). Study enrollment took place in April 2013, and the eight-week program was carried out from the end of April to the end of June 2013. After the eight-week program concluded, participants completed a post-program assessment to assess potential changes in mental well-being and mindfulness.

### 2.2. Telephone-Adapted Mindfulness-Based Stress Reduction Program

The standard MBSR program is an eight-week program that meets in-person once a week for 2.5 h, and includes a full day of practice between the sixth and seventh weeks. Participants are also instructed to practice at home for 45 min each day using pre-recorded guided meditations. Adaptations to the standard in-person MBSR program were made in order to deliver the program by telephone. First, each session in the adapted program was shortened to 90 min, and the full-day retreat was removed. Second, participants were provided with extensive printed materials as a guide for each session, including a program binder with written handouts and detailed illustrations for yoga poses. Similar to the standard program, all participants received a CD with pre-recorded tracks to support home practice.

The telephone-adapted mindfulness-based stress reduction (MBSR) program was led by an instructor with extensive experience in teaching MBSR across diverse settings. At study enrollment, participants were provided with the program materials, which included the program binder and CD with pre-recorded guided meditations. The sequence of practices was the same as the standard MBSR program, including the body scan awareness practice, sitting meditation with awareness of breath, mindful yoga, mindful walking, and loving-kindness meditation. The program was conducted via weekly group sessions over the course of eight weeks and was held on a weekday evening. Participants received instructions on how to call in to the group teleconference using a specific number and access code. 

During the program, participants were taught mindfulness techniques including the body scan, awareness of breathing, mindful yoga, and sitting meditation (see Table 1 for a brief description of each session’s content and activities). Class time was divided between meditation practice, group discussions, and mindfulness skill-building activities. Class activities were designed to (a) enhance awareness of one’s body and mind, (b) teach participants to replace automatic reactions with consciously chosen responses, and (c) bring greater awareness and skill to interpersonal communication. Group discussions focused on participants’ experiences with meditation practices and on the application of mindfulness in day-to-day life. The instructor provided homework assignments for each week, and participants were instructed regarding the use of the audio recordings to support home practice.

### 2.3. Measures

#### 2.3.1. Demographic Characteristics

Participant characteristics including age, sex, race and ethnicity, marital status, highest level of education and occupational category were assessed at study entry.

#### 2.3.2. Feasibility and Acceptability

Feasibility was assessed via rates of post-enrollment drop-out and session completion. Acceptability was explored using participants’ feedback captured on the post-program assessment. Specifically, participants responded to an open-ended item requesting comments about the program and their experience as a participant in this program. Responses were organized thematically to provide descriptive information about program acceptability.

#### 2.3.3. Psychological Distress

Psychological distress was assessed using the Brief Symptom Inventory-18 (BSI-18 [27]), which is an 18-item instrument adapted from the original Symptom Checklist (SCL-90-R [28]). For each item, participants were asked to report the extent to which they have been “distressed or bothered” by that symptom in the prior seven days. Response options ranged from “0 = not at all” to “4 = extremely.” Scores were then summed across each of three subscales: somatization, depression, and anxiety. The three subscale scores were combined to form the Global Severity Index (GSI) composite score, which provides an overall score of psychological distress. Following scoring instructions, raw scores were converted to gender-specific T-scores based on community norms. The BSI-18 is highly reliable, and internal consistency of each subscale ranged from 0.74 to 0.90. The BSI-18 has been widely utilized in prior research including studies of mindfulness training with healthcare professionals [29].

#### 2.3.4. Mindfulness and Self-Compassion

Two well-established measures were used to assess mindfulness and self-compassion: the Five Facet Mindfulness Questionnaire (FFMQ [30]) and the Self-Compassion Scale (SCS [17,31]). The 39-item FFMQ measures five aspects of mindfulness: observing, describing, acting with awareness, non-judging of inner experience, and non-reactivity to inner experience. The observing subscale is comprised of eight items and assesses attention to or observation of present-moment experiences, both internal (e.g., emotions, thoughts) and external (e.g., sounds, smells). Describing is comprised of eight items and reflects the ability to label or express experiences in words. Acting with awareness (eight items) measures one’s ability to be present in one’s activities, rather than behaving automatically while one’s attention wanders and focuses elsewhere. Non-judging of inner experience is measured using eight items that assess one’s ability to accept thoughts and emotions without attaching judgement (e.g., “good” or “bad” thoughts). Non-reactivity to inner experience includes seven items and measures the ability to experience unpleasant thoughts or emotions without reacting with maladaptive, counter-productive thoughts or behaviors. Participants respond to each item on a 5-point Likert-type scale by rating how often each aspect is true of them, ranging from “1 = Never or very rarely true” to “5 = Very often or always true.” Scores are summed across each subscale item to compute a subscale score. The scale has demonstrated good test-retest reliability and construct validity [32] and the internal consistency of each subscale was high, with Cronbach’s α coefficients ranging from 0.83 to 0.92.

The 26-item Self-Compassion Scale (SCS [17,31]) is widely used to assess self-compassion in mindfulness-based intervention research. The measure has six subscales: self-kindness, self-judgment, common humanity, isolation, mindfulness, and over-identification. Five items assess self-kindness or the capacity to be understanding toward ourselves and to treat ourselves with compassion and kindness (e.g., “When I’m going through a very hard time, I give myself the caring and tenderness I need”). Self-judgment (five items) involves being critical of one’s shortcomings and flaws (e.g., “I’m disapproving and judgmental about my own flaws and inadequacies”). The common humanity and isolation subscales are measured using four items each and assess the extent to which an individual perceives difficulties and suffering as part of a shared human experience (e.g., “When I’m down and out, I remind myself that there are lots of other people in the world feeling like I am”) or an experience that is specific to that individual alone (e.g., “When I fail at something that’s important to me, I tend to feel alone in my failure”). The mindfulness subscale is assessed using four items that measure the ability to observe one’s thoughts and feelings in a balanced way (e.g., “When something painful happens, I try to take a balanced view of the situation”). The over-identification subscale (four items) captures the tendency to over-identify with and exaggerate negative thoughts and feelings (e.g., “When I fail at something important to me, I become consumed by feelings of inadequacy”). The SCS has demonstrated robust psychometric properties. In the present study, Cronbach’s α ranged from 0.81 to 0.95 across the six subscales.

### 2.4. Analytic Plan

To assess feasibility, percentages were used to describe post-enrollment drop-out rates and session completion. Participants’ feedback and comments from the post-program survey informed acceptability and opportunities for program refinement. Per recent recommendations for the reporting of preliminary data [33], means and 95% confidence intervals (CI) were used to characterize participants’ levels of psychological distress, mindfulness, and self-compassion at baseline and post-program. Inter-individual differences were calculated between baseline and follow-up, and confidence intervals were obtained around these inter-individual differences. Standardized effect sizes were also calculated using Cohen’s *d* statistic, with *d* = 0.2 representing a ‘small’ effect size, *d* = 0.5 representing a ‘medium’ effect size, and *d* = 0.8 representing a ‘large’ effect size [34]. Analyses were performed using SPSS version 26 (Armonk, NY, USA). 

## 3. Results

### 3.1. Participant Characteristics

Twelve individuals contacted the study team about participating in the program; eleven consented, but one declined to participate in the intervention due to the time commitment involved. The demographic characteristics of the study sample are reported in Table 2. Participants were on average 50 years of age (SD = 13.9 years). The majority were female (72.7%), married (63.6%), and non-Hispanic white (63.6%). Over one third of participants (36.4%) had received a college or post-graduate degree. About one half of the sample were clinical staff (i.e., staff with direct patient contact, such as physicians, registered nurses, clinical research associates, etc.), while the remaining were support staff (such as those in administrative, business, facility support, or volunteer roles).

### 3.2. Feasibility and Acceptability

Among the 11 participants, one (9%) dropped out after the first week due to time constraints. The remaining ten participants completed, on average, 6.2 sessions in the eight-week program (range = 3-8 sessions). These rates of drop-out and average session completion are comparable to a previously published telephone-adapted MBSR study (among participants awaiting kidney transplant) [35]. 

In our study, 90% (*n* = 9) of the participants completed at least half of the program (≥4 sessions), which is the suggested minimal number of MBSR sessions needed to observe a change in distress [36]. Seven participants completed six or more sessions (75% of the program), which prior studies have considered a minimal number of sessions for MBSR to count as completion [37,38,39,40,41]. This percentage is also in line with rates considered as evidence of feasibility in other studies [42]. Two participants completed four or five sessions (50% or more of the program), and one participant completed three sessions. Thus, overall retention and completion rates in the current study are similar to rates reported in prior published studies and provide preliminary evidence of feasibility.

Of the ten participants, nine completed the post-program assessment in which they were asked to provide open-ended feedback and comments about the program. Participants’ responses indicated positive feedback. They generally reported enjoying the content and delivery, and perceived that the program had positive impacts on daily life.

“*I enjoyed every week of this program. I did not miss any classes. The mindfulness class has opened my eyes. In time I think it will be very beneficial in my life.*”

—ID #105

“*[The instructor] was wonderful and very calm. Perfect for this program. I feel blessed to have been able to take this class. Thank you!*”

—ID #110

“*Interesting and beneficial program.*”

—ID #112

Participant comments also provided guidance for how to improve program delivery in future versions. For example, participants noted that offering video-based materials, having shorter sessions over the phone, or using a hybrid format with at least one session in person could be advantageous.

“*It would be helpful to have a DVD for the yoga. Pictures were not adequate.*”

—ID #109

“*Might be a good idea to have participants meet at least once prior to the program. Send a CD or DVD to show yoga positions-difficult to just do orally.*”

—ID #112

“*I feel the sessions would have been more beneficial if they were in person. I think there would have been more interaction/sharing with [the instructor] and the other group members if we had met in person. An hour and a half is a long time to be on the phone. Thank you.*”

—ID #103

### 3.3. Psychosocial Outcomes

Variable means, 95% confidence intervals (CI), and standardized pre-post effect sizes for each measure are presented in Table 3.

Consistent with prior research, medium to large effect sizes were observed for psychological distress (Table 3). Specifically, participants reported reductions in depression (mean difference = −9.4, 95% CI −18.2 to −0.6). Overall psychological distress also decreased from pre- to post-program (mean difference = −9.4, 95% CI −15.9 to −2.9). Similarly, we observed reductions in anxiety (mean difference = −8.2, 95% CI −17.3 to 1.0) and somatization (mean difference = −5.0, 95% CI −10.4 to 0.5) with effect sizes in the medium range (*d* = 0.68 to 0.70).

With respect to mindfulness as measured by the FFMQ, improvements were observed in non-reactivity, with a mean increase of 2.3 (95% CI −0.9 to 5.5) and a medium effect size (*d* = 0.55). Small to medium effects emerged for observing and acting with awareness, as well as for the overall FFMQ total mindfulness score. Finally, for self-compassion, participants reported increases in common humanity (mean difference = 2.9, 95% CI 0.5 to 5.4) with a very large effect size (*d* = 0.91) (Table 3). Other than modest changes in self-judgment (mean difference = 1.8, 95% CI −1.8 to 5.5), which showed a small to medium effect (*d* = 0.38), there was relatively limited change on the remaining self-compassion subscales.

## 4. Discussion

Our findings suggest that a telephone-based format may be a feasible mode of delivery for an eight-session MBSR program for healthcare employees. Given the high rates of attrition observed among in-person MBSR programs for healthcare employees [21,23], a telephone-based format may offer key advantages such as greater ease of attendance and a sense of anonymity, which may help increase the program’s acceptability for this population. As hypothesized, qualitative feedback on the program indicated that participants found the classes to be enjoyable and useful. However, participants suggested some improvements to be considered, such as using more visual Supplemental Materials (e.g., video/DVD) and incorporating some in-person features to the program, as not all participants would have preferred the telephone as the sole modality of the intervention. Future iterations of a telephone-based MBSR program may offer a hybrid format (i.e., with selected sessions in-person) in order to provide greater guidance for home practice and to enhance participant interactions. 

Although we had a small sample size, our preliminary outcome measures showed evidence of positive psychosocial changes consistent with the literature [21,22,23,24]. We observed positive changes in depressive symptoms and overall distress, which offers initial support for the premise that a telephone-based MBSR program can be beneficial for alleviating mood symptoms in healthcare professionals. The effect sizes for many measures were large, but in this small pilot trial the confidence intervals were large as well. Thus, future studies with larger samples are required to confirm whether a telephone-adapted MBSR program can lead to significant improvements in these domains. Nevertheless, these findings add to the growing literature that telephone adaptions of MBSR may be promising for improving mental health outcomes [35]. 

While all mindfulness (FFMQ) subscale scores showed an increase over time (with small to medium effect sizes), the largest effect size was observed for non-reactivity, which is the ability to allow one’s thoughts and feelings to come and go without being carried away by them [30,43]. This finding is consistent with other mindfulness studies, which have reported that non-reactivity shows among the largest increases in subscale scores after MBSR participation [44,45,46]. Furthermore, this subscale is frequently the strongest correlate of positive psychological outcomes in other mindfulness studies [47,48,49,50]. Interestingly, acting with awareness showed the largest increase in mean scores in our study. This finding contrasts with what is observed among in-person mindfulness interventions in populations not specific to healthcare [46], and could suggest that changes in acting with awareness may be a unique process either within healthcare workers or with the telephone format. Taken together, non-reactivity and acting with awareness may be particularly beneficial skills to build among healthcare employees, in light of the need to remain calm, attentive, and composed in high stress environments. Given that the various mindfulness subscales can differentially relate to psychosocial outcomes [50,51], future research should more deeply explore how mindfulness skills can be enhanced in telephone-based MBSR programs, as well as within healthcare employees.

An interesting observation that emerged was that common humanity seemed to increase the most among the self-compassion subscales, and it did so to a large effect. Common humanity is a sense of positive connection to those around you created through the recognition that suffering and personal inadequacy are shared human experiences [17]. Importantly, our findings suggest that telephone-based MBSR still fostered this sense of interconnectedness, despite the absence of in-person sessions. Common humanity may have been enhanced via compassion-based MBSR practices such as the lovingkindness meditation [52], which is a contemplative practice used to evoke feelings of love, warmth, and care towards the self and others around us [53]. Common humanity may have also been encouraged through the group-based format and group discussions, which could have underscored a sense of common purpose among the healthcare employees (e.g., a sense of “we’re all in this together.”) Indeed, healthcare employees who experience higher levels of common humanity also report better psychosocial outcomes, including greater happiness, lower distress, and lower burnout [54,55]. Future work should continue to explore the role of common humanity in populations that may experience feelings of isolation and disconnection, and how various modes of MBSR delivery can change this domain. 

### 4.1. Limitations

Our study had several limitations. First, our sample size was small and was not powered to find effects; thus, our results should be interpreted with caution. Larger sample sizes will be able to help provide additional evidence for the feasibility of a telephone adapted program. Second, while one strength of our sample was that it included a diversity of professional roles, it is possible that needs, preferences, and outcomes may differ among those in clinical versus support roles. Our sample was also largely comprised of female participants; thus, gender may indirectly impact our findings. Similarly, we did not measure previous experience with mindfulness, which could be important to include as a covariate in future studies. Future studies may wish to explore these questions further. Third, our study design was non-randomized; thus, direct causal interpretations should not be made. The absence of a control group limits our ability to provide comparisons for how psychological distress, mindfulness, or self-compassion may have changed naturally over time. Despite these limitations, the present study offers important suggestions for how MBSR can be adapted for telephone delivery, as this mode of delivery may facilitate increased participation given the greater ability to accommodate healthcare employees’ schedules and needs.

### 4.2. Future Directions

Future research can build upon our study in several ways. To address our limitations, larger and more diverse samples (regarding gender, race, etc.) as well as a randomized study design (including a control group and/or the traditional in-person MBSR program) should be utilized. This would also allow for additional informative analyses such as whether certain demographic characteristics such as gender, education level, or profession are associated with intervention participation. In addition, future adaptations could include greater use of visual Supplemental Materials (e.g., videos) to support home practice, and researchers may wish to gather more detailed data on practice adherence. If demonstrated to be effective, the application of a telephone-based MBSR program could be extended and evaluated among other populations for which in-person programs may be challenging, such as rural or low socio-economic populations, or those with severe illness. Furthermore, given that our sample did not report clinical levels of distress at baseline, future studies may wish to explore the application of a telephone-based MBSR program in a highly distressed sample. Finally, larger iterations of this study could also explore potential mediators, such as which mindfulness skills are key for improving emotional outcomes among healthcare employees.

### 4.3. Conclusions

The purpose of the current study was to adapt and deliver a telephone-based format of MBSR among healthcare employees in order to assess its feasibility and acceptability. Our findings indicate that the program was feasible and largely acceptable; however, additional research is needed to optimize this mode of delivery. Nevertheless, our preliminary outcomes suggest that a telephone-adapted MBSR program may show promise for improving psychosocial outcomes, especially distress, depression, and a sense of common humanity among healthcare employees. Overall, facilitating the delivery of evidence-based MBSR programs to healthcare employees, a population that experiences high stress and professional burnout, addresses a significant need and may yield psychosocial benefits.

## Figures and Tables

**Table 1:** Program Curriculum.

Session	Topic	Activities
1	- Introduction to mindfulness - Attitudinal foundations	- Relaxing sighs - Body scan meditation
2	- Physiology of stress - Perception and creative responding - Cultivating mindfulness in daily activities	- Sitting meditation: Awareness of breath
3	- Pleasure and power in being present	- Body scan - Meditation in motion: Yoga
4	- Awareness of stress reactivity: sensations, emotions, and thoughts	- Mindful yoga: Cultivating strength, balance, and flexibility
5	- Responding vs. reacting to stress: The role of mindfulness - Moving from habitual behaviors to choosing more effective responses	- Sitting meditation: Expanded awareness
6	- Interpersonal communication skills: A mindful approach	- Sitting meditation: Expanded awareness
7	- Mindful communication continued - Cultivating kindness and compassion for self and others	- Loving kindness meditation
8	- Beyond eight weeks: Mindfulness resources - Continuing to cultivate mindfulness in day-to-day life - Meditation on honorable closure	- Reflection on eight weeks - Setting intentions for moving forward

**Table 2:** Participant Characteristics (*n* = 11).

Variable	No. of Participants (%)	Mean (SD)
Age (years)		49.9 (13.9)
Gender		
Male	3 (27.3%)	
Female	8 (72.7%)	
Race/Ethnicity		
Non-Hispanic White	7 (63.6%)	
Non-Hispanic Black	1 (9.1%)	
Non-Hispanic Asian	1 (9.1%)	
Hispanic White	1 (9.1%)	
Unreported	1 (9.1%)	
Marital status		
Single	4 (36.4%)	
Married/living as married	7 (63.6%)	
Education		
High school/vocational school	3 (27.3%)	
Some college	4 (36.4%)	
College /Post-grad degree	4 (36.4%)	
Occupational Category		
Clinician/clinical staff	5 (45.5%)	
Support staff	6 (54.4%)	

**Table 3:** Means, Differences, and 95% Confidence Intervals (CI) on Outcome Measures (*n* = 9).

	Baseline M (95% CI)	Post-MBSR M (95% CI)	Difference M (95% CI)	Effect Size
BSI-18				
Somatization	54.1 (47.0, 61.2)	49.1 (42.9, 55.4)	−5.0 (−10.4, 0.5)	*d* = 0.70
Depression	57.1 (48.1, 66.1)	47.7 (42.4, 53.0)	−9.4 (−18.2, −0.6)	*d* = 0.82
Anxiety	56.3 (46.3, 66.3)	48.1 (44.8, 51.5)	−8.2 (−17.3, 1.0)	*d* = 0.68
Global Severity Index	58.2 (52.2, 64.2)	48.8 (47.0, 50.7)	−9.4 (−15.9, −2.9)	*d* = 1.11
FFMQ				
Observing	20.2 (16.7, 23.7)	22.4 (19.1, 25.8)	2.2 (−1.6, 6.0)	*d* = 0.45
Describing	25.2 (19.7, 31.8)	26.2 (21.7, 30.7)	1.0 (−1.9, 3.9)	*d* = 0.27
Acting with awareness	25.4 (19.2, 31.7)	28.5 (25.1, 31.9)	3.1 (−2.9, 9.0)	*d* = 0.40
Non-judging	26.9 (21.2, 32.6)	28.8 (24.7, 32.9)	1.9 (−6.2, 9.9)	*d* = 0.18
Non-reactivity	16.4 (12.8, 20.1)	18.8 (15.9, 21.7)	2.3 (−0.9, 5.5)	*d* = 0.55
Total mindfulness	111.6 (97.2, 125.9)	122.0 (109.2, 134.8)	10.4 (−5.8, 26.7)	*d* = 0.49
SCS				
Self-kindness	14.2 (10.4, 18.0)	13.8 (11.4, 16.2)	−0.4 (−4.1, 3.3)	*d* = 0.08
Self-judgment	17.3 (14.8, 19.9)	19.2 (17.2, 21.2)	1.8 (−1.8, 5.5)	*d* = 0.38
Common humanity	10.6 (7.0, 14.1)	13.5 (10.8, 16.2)	2.9 (0.5, 5.4)	*d* = 0.91
Isolation	13.7 (9.5, 17.8)	13.0 (10.3, 15.7)	−0.7 (−4.8, 3.5)	*d* = 0.13
Mindfulness	12.7 (8.9, 16.4)	13.3 (11.4, 15.2)	0.7 (−2.2, 3.5)	*d* = 0.19
Over-identification	12.6 (9.8, 15.3)	13.4 (11.3, 15.5)	0.8 (−2.6, 4.3)	*d* = 0.18
Total self-compassion	71.9 (53.8, 90.0)	73.2 (63.8, 82.7)	1.4 (−16.6, 19.4)	*d* = 0.06

## Data Availability

Data may be made available by the principal investigator upon reasonable request.
